# An immune-related gene prognostic risk index for pancreatic adenocarcinoma

**DOI:** 10.3389/fimmu.2022.945878

**Published:** 2022-07-26

**Authors:** Yang Su, Ruoshan Qi, Lanying Li, Xu Wang, Sijin Li, Xuan Zhao, Rui Hou, Wen Ma, Dan Liu, Junnian Zheng, Ming Shi

**Affiliations:** ^1^ Jiangsu Center for the Collaboration and Innovation of Cancer Biotherapy, Cancer Institute, Xuzhou Medical University, Xuzhou, China; ^2^ College of Pharmacy, Xuzhou Medical University, Xuzhou, China

**Keywords:** pancreatic adenocarcinoma, immune gene, prognosis, immunotherapy, CTLA4

## Abstract

**Objective:**

Our goal is to construct an immune-related gene prognostic risk index (IRGPRI) for pancreatic adenocarcinoma (PAAD), and to clarify the immune and molecular features in IRGPRI-defined PAAD subgroups and the benefit of immune checkpoint inhibitors (ICIs) therapy.

**Method:**

Through differential gene expression analysis, weighted gene co-expression network analysis (WGCNA), and univariate Cox regression analysis, 16 immune-related hub genes were identified using the Cancer Genome Atlas (TCGA) PAAD dataset (n = 182) and immune gene set. From these genes, we constructed an IRGPRI with the Cox regression method and the IRGPRI was verified based on the Gene Expression Omnibus (GEO) dataset (n = 45). Then, we analyzed the immune and molecular features and the benefit of ICI therapy in IRGPRI-defined subgroups.

**Results:**

Five genes, including *S100A16*, *CD40*, *VCAM1*, *TNFRSF4* and *TRAF1* were used to construct IRGPRI. As with the results of the GEO cohort, the overall survival (OS) was more favorable in low IRGPRI patients versus high IRGPRI patients. The composite results pointed out that low IRGPRI was associated with immune response-related pathways, high level of CTLA4, low KRAS and TP53 mutation rate, more infiltration of activated memory CD4^+^ T cells, CD8^+^ T cells, and more benefits from ICIs therapy. In comparison, high IRGPRI was associated with cancer-related pathways, low expression of CTLA4, high KRAS and TP53 mutation rate, more infiltration of M2 macrophages, and less benefit from ICIs therapies.

**Conclusion:**

This IRGPRI is an encouraging biomarker to define the prognosis, immune and molecular features, and benefits from ICIs treatments in PAAD.

## Introduction

Pancreatic adenocarcinoma (PAAD) is a high-graded neoplasm of digestive system, with a 5-year survival rate of lower than 10% (1). PAAD is predicted to become the second-leading cause of cancer death by 2030 (2, 3). Owing to lifestyle changes, the global incidence of PAAD is expected to increase (4). In clinical practice, histological grading, tumor staging and molecular classification may be employed to assess in the prognosis of PAAD patients. However, these clinicopathological features generally cannot provide accurate prognostic information for patients (5). Some inflammatory molecules are involved in the prognosis of PAAD patients; however, their sensitivity and specificity are not robust enough (6). Currently, some researches pay attention to the immune-related gene signatures in the prognosis of PAAD (7–9). For example, Zhang Q’s team built a prognostic model of PAAD using 3 lncRNA pairs (10). Bu F and his colleagues construct a prognostic model of PAAD using 18 immune-related gene pairs (11). Nevertheless, few studies tried to build the prognostic model of PAAD based on the immune-related central genes. Here, we screened immune-related central genes associated with the patient prognosis through weighted gene co-expression network analysis (WGCNA). Meanwhile, few studies pay attention to immune features and immunotherapy of PAAD at the same time.

The treatment of PAAD remains a major challenge, and surgery is an option of the highest priority. However, only 15~20% patients are suitable for resection, and 80% of those who undergo surgery will recur (12). Radiotherapy and chemotherapy have been shown to benefit patients with PAAD and improve the overall survival; however, the survival rate remains low (13). There is no therapeutic drug that can provide nonsurgical candidates with long-term benefits (13). Immunotherapy is an exciting new anticancer therapy that activates the immune system to identify tumor-specific antigens (14, 15). Clinical trials of PAAD have showed that immunotherapy has a good application prospect in the treatment of PAAD (16). In addition, resistant individuals are better candidates for immunotherapy (17). Immune checkpoint inhibitor (ICI) therapy, such as those targeting cytotoxic T lymphocyte-associated protein 4 (CTLA4), programmed death-ligand 1 (PD-L1) and programmed death 1 (PD1), have been shown to be significantly beneficial for the survival versus traditional therapies (1–5). For PAAD, anti-CTLA4 therapy leads to an enhanced anti-tumor immune response (18, 19). However, a variety of factors may affect the effectiveness of immunotherapy, such as the tumor microenvironment (TME), and few immunogene-based biomarkers are good predictors of the patient prognosis. Identifying potential prognostic markers associated with treatment benefits is conducive to the individualize immunotherapy of PAAD patients. Therefore, it is urgent to identify indicators that can predict the prognosis and immunotherapeutic effect of PAAD.

Here, we aimed to explore prognostic markers for PAAD that could predict the results of traditional therapy and suggest the value of immunotherapy. By focusing on all immune-related genes (IRGs) in PAAD transcriptome data, the present study was designed to screen IRGs associated with the patient prognosis through WGCNA, and construct the IRGPRI. Subsequently, we described the molecular and immunological characteristics of IRGPRI and detected its ability to predict the patient prognosis and ICI therapy efficacy. The results suggest that IRGPRI is an encouraging prognostic biomarker.

## Methods

### Datasets and patients

The RNA sequence data (RNA-seq data) and clinicopathological information of 182 PAAD samples (178 cancer samples vs. 4 para-cancer samples) were obtained from the TCGA database (https://portal.gdc.cancer.gov/). Additionally, RNA-seq data of 45 PAAD samples (GSE28735) and their survival information were obtained from the GEO database. Expression data in human renal cell carcinoma samples (GSE67501) and metastatic melanoma (GSE115821) from patients who did or did not respond to ICI therapy were also obtained from the GEO database. The IRG list was derived from the ImmPort (https://www.immport.org/shared/home) databases and InnateDB (https://www.innatedb.ca/).

### Identification of immune-related hub genes

According to the RNA-seq data of PAAD samples (178 cancer samples vs. 4 para-cancer samples) derived from TCGA, lists of genes in different expressions (p < 0.05, |log2FC| > 1) were determined with the limma package of R. From InnateDB and ImmPort, we obtained the immune-related gene lists. IRGs in different expressions were obtained and analyzed with Gene Ontology (GO) and Kyoto Encyclopedia of Genes and Genomes (KEGG) analyses by using the clusterProfiler package of R.

Then, hub genes were determined by WGCNA. First, calculated the Pearson correlation coefficient between two genes, according to the expression data to design the similarity matrix, and then using a network type of a signed and soft threshold β = 6 to convert into an adjacency matrix, followed by transformation into a topological matrix by using the topological overlap measure (TOM) indicating the degree of correlation between genes. 1-TOM was selected as the distance to cluster the genes, and then a dynamic pruning tree was constructed to determine the modules. In the end, three modules were identified by assigning the merging threshold function as 0.3. Based on the genes of notably related modules (the turquoise and blue modules), the network was constructed by between-gene edges at the weight of more than 0.3. The genes in turquoise modules were used for subsequent analyses, of which 16 significantly survival-associated IRGs were used for further analyses (p < 0.05, log-rank test).

### Construction and verification of the IRGPRI

Among 16 immune-related hub genes, based on multivariate Cox regression analysis, the five genes that had a significant effect on OS were employed to construct an IRGPRI. In the Cox model, we calculated the IRGPRI of each sample as per the formula: IRGPRI = [Expression level (certain genes) × gene coefficient]. The prognostic ability of the IRGPRI was assessed by K-M survival curve and log-rank test with both GEO and TCGA cohorts. Univariate and multivariate Cox regression analyses were performed to verify the independent prognostic value of IRGPRI.

### Thorough assessment of molecular and immunologic features and ICI therapy in high IRGPRI and low IRGPRI groups

For signaling pathway analysis, limma package of R was used for analyzing low IRGPRI (n = 89) and high IRGPRI (n = 88) samples by differential expression analysis of all genes. The clusterProfiler package of R (p < 0.05) was used to preform gene set enrichment analysis (GSEA) method on GO and HALLMARK gene sets, in order to identify the signaling pathways where genes in different expressions were implicated. GSVA package of R was utilized for single sample GSEA (ssGSEA) analysis of several typical gene sets. For gene mutation analysis, genetic alteration data were downloaded from the TCGA database. Then, we performed correlation analyses to analyze the correlation between IRGPRI and the expression of CTLA4 and PD-L1 (CD274).

To determine immune features of PAAD samples, their expressions were input into CIBERSORT (https://cibersort.stanford.edu/) with 1,000 iterations to calculate the relative percentage of 22 classes of immune cells. Next, we made a comparison of the obtained percentage and clinicopathological factors between two IRGPRI subgroups, and assessed the results by means of a landscape map.

### Statistical analysis

Using an independent t-test, we carried out comparison of continuous variables between high IRGPRI and low IRGPRI groups. Categorical data were analyzed by the chi-square test. Beyond that, univariate survival analysis was completed by Kaplan-Meier survival analysis and the log-rank test. Multivariate survival analysis was conducted in the Cox regression model. A two-sided p-value < 0.05 was accepted as statistically significant differences.

## Results

### Immune-related hub genes

By differential expression analysis (178 cancer samples vs. 4 normal samples), 1672 genes in different expressions were obtained (**Figure S1A**). Through intersection of these genes with IRGs from InnateDB and ImmPort, 245 IRGs in different expressions were identified (**Figure S1B**). There was a remarkable association of 245 genes in different expressions with 1058 GO terms and 67 KEGG pathways, as indicated by functional enrichment analysis (**Table S1**). Top 8 GO terms and KEGG pathways are provided in **Figure S1C** and **S1D**.

**Figure 1:**
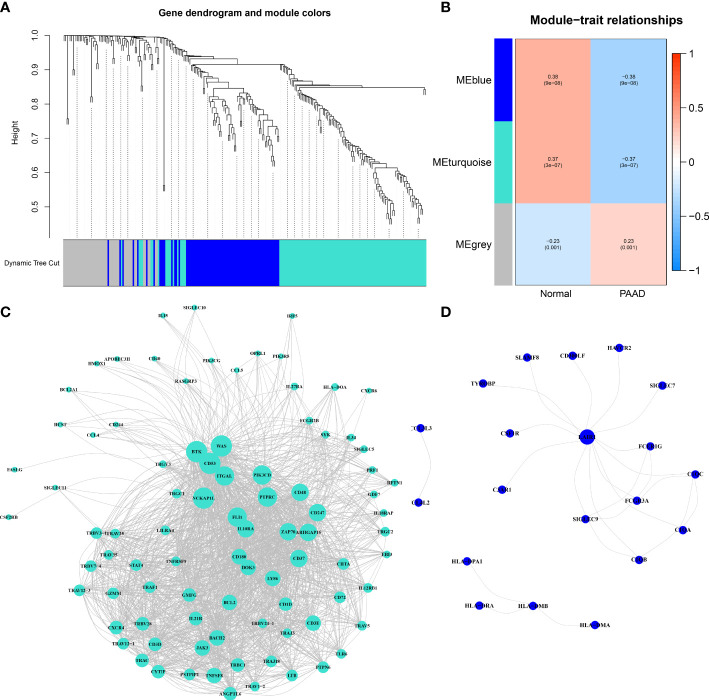
Immune-related hub genes. **(A)** Weighted gene coexpression network analysis (WGCNA) of immune-related differentially expressed genes with a soft threshold β = 6. **(B)** Gene modules related to PAAD obtained by WGCNA. **(C)** The network of the genes in the turquoise module (weight of edge > 0.3). **(D)** The network of the genes in the blue module (weight of edge > 0.3).

**Figure 2:**
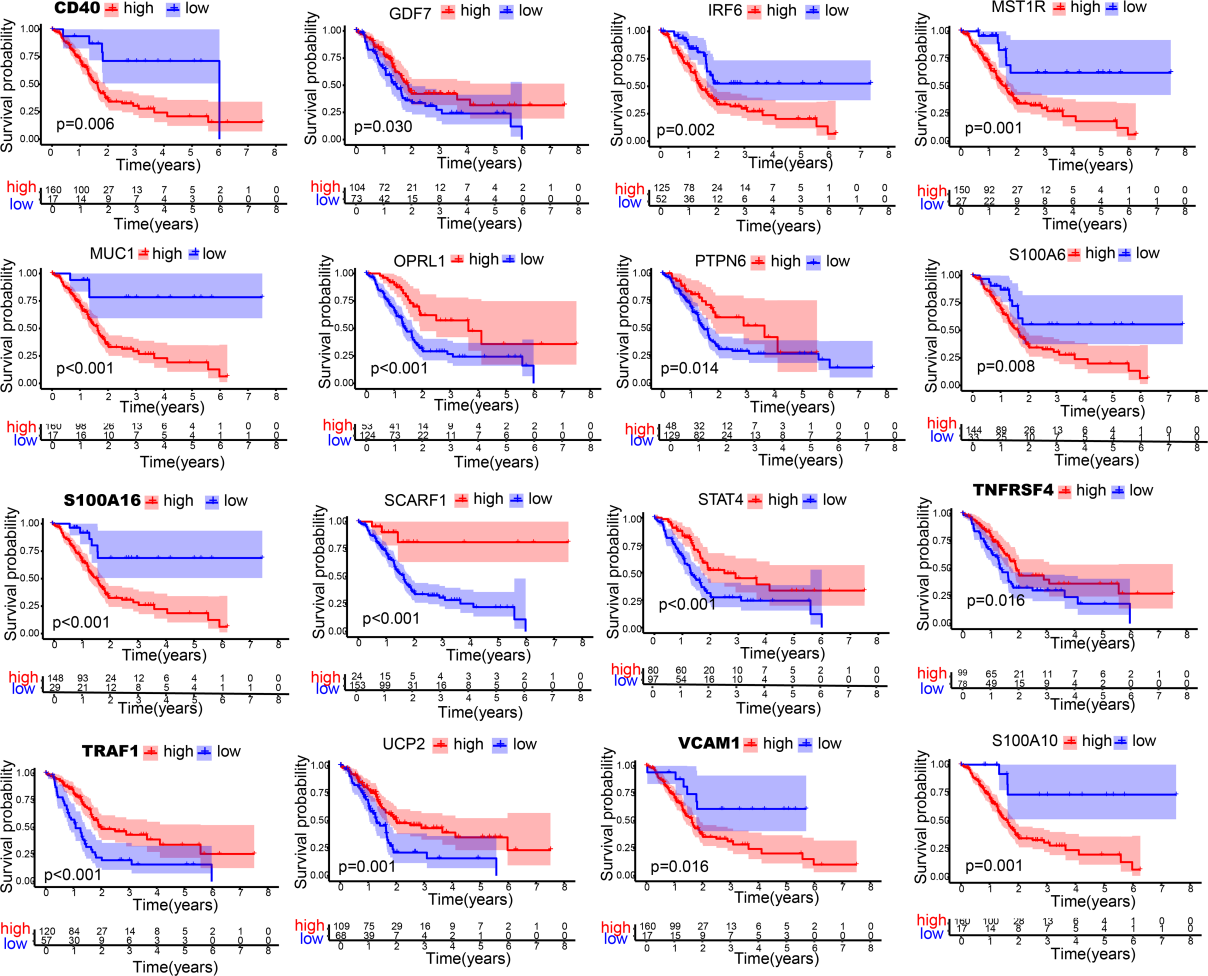
Kaplan-Meier curves of 16 immune-related hub genes. Kaplan-Meier survival analysis of 16 immune-related genes in TCGA cohort.

**Figure 3:**
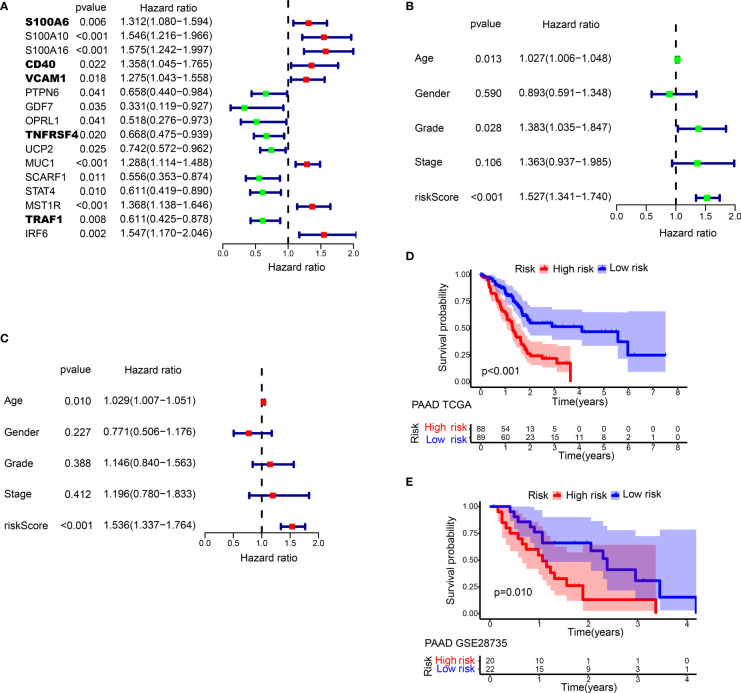
Prognostic analysis of different IRGPRI subgroups. **(A)** Univariate Cox analysis of 16 immune-related hub genes. **(B)** Univariate Cox analysis of clinicopathological factors and the IRGPRI score. **(C)** Multivariate Cox analysis. **(D)** Kaplan-Meier survival analysis of the IRGPRI subgroups in the TCGA cohort. **(E)** Kaplan-Meier survival analysis of the IRGPRI subgroups in the GEO cohort (GSE28735).

To determine the immune-related hub genes, we performed WGCNA analysis on the candidate genes (n = 245). A negative correlation was observed between the logarithm log(k) of the node with connectivity K and the logarithm log (P (k)) of the probability of the node. According to the scale-free network, the best soft-thresholding power was 6 (**Figure S2**). According to the best soft-thresholding power and the average linkage hierarchical clustering, 3 modules were identified (**Figures 1A**, **B**), to which 245 genes were assigned. Based on the Pierson correlation coefficient between the module and sample characteristics of each module, turquoise and blue modules were strongly associated with PAAD. There were 24 edges and 20 genes for the blue module, 1489 edges and 88 genes for the turquoise module of the networks with a threshold weight of more than 0.3 (**Figures 1C**, **D**). Thus, the genes in the turquoise module were used for further analyses. We obtained all 116 genes in the turquoise module. We determined that the expression level of 16 immune-related hub genes of them was strongly correlated with OS of PAAD patients, as shown in **Figure 2** and **Figure 3A**.

### Survival outcomes in different IRGPRI groups

The prognostic index was constructed for each cancer sample calculated by the coefficient in **Table S2**.

In univariate Cox regression analysis, IRGPRI, grade, and age were notably associated with the prognosis of PAAD (**Figure 3B**). Later, IRGPRI was proven to be an independent prognostic factor by multivariate Cox regression analysis, (**Figure 3C** and **Table S3**).

With the cutoff value of the median IRGPRI, low IRGPRI patients achieved better OS than high IRGPRI patients based on the TCGA dataset (*p <* 0.001, log-rank test) (**Figure 3D**). The roles of IRGPRI were then validated by the GSE28735 PAAD dataset (n = 45). In **Figure 3E**, patients in the low IRGPRI subgroup achieved a notably favorable prognosis versus high IRGPRI subgroup (*p =* 0.010, log-rank test).

### Molecular features in different IRGPRI subgroups

**Figure 4:**
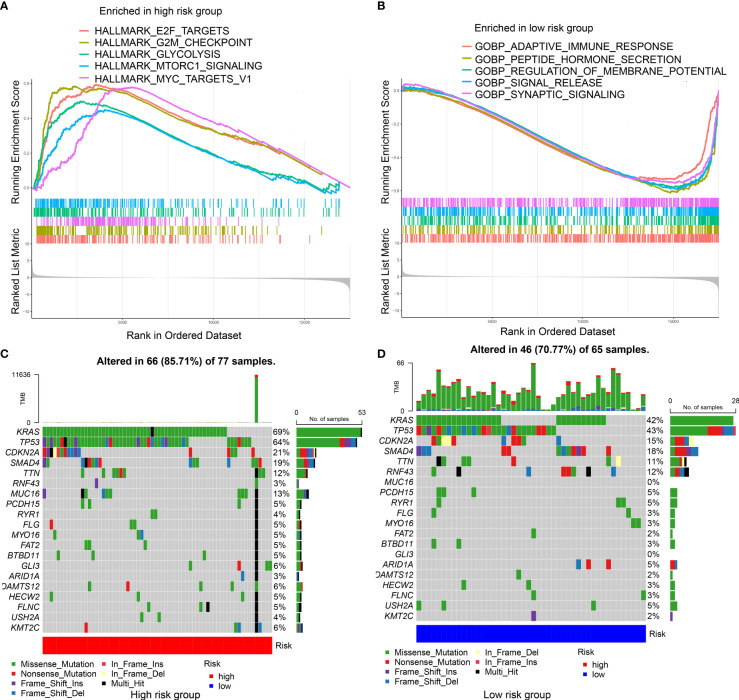
Molecular characteristics of different IRGPRI subgroups. **(A)** Gene sets enriched in IRGPRI-high group. **(B)** Gene sets enriched in IRGPRI-low group. **(C, D)** Significantly mutated genes in the mutated PAAD samples of IRGPRI-high group **(C)** and IRGPRI-low group **(D)**. Mutated genes (rows) are ordered by mutation rate; samples (columns) are arranged to emphasize mutual exclusivity among mutations. The right shows mutation percentage, and the top shows the overall number of mutations. The color-coding indicates the mutation type.

Enriched gene sets in different IRGPRI subgroups were determined by GSEA. Some cancer-related pathways were observed in high IRGPRI samples (**Figure 4A**), while enriched gene sets of low IRGPRI samples were identified in some immune response-related pathways (**Figure 4B**).

Next, gene mutation analysis was performed to obtain further biological information on the immunological nature of the IRGPRI subgroups. Missense variation was identified as the most common mutation, followed by nonsense and frameshift insertion. Top 20 genes with the greatest mutation rate were then determined in the IRGPRI subgroups. In both groups, the mutation rates of KRAS, TP53, CDKN2A, and SMAD4 were all greater than 15%. The high IRGPRI subgroup showed more mutations of KRAS, TP53, and MUC16 genes (**Figure 4C**), while the low IRGPRI subgroup had more mutation of RNF43 genes (**Figure 4D**).

**Figure 5:**
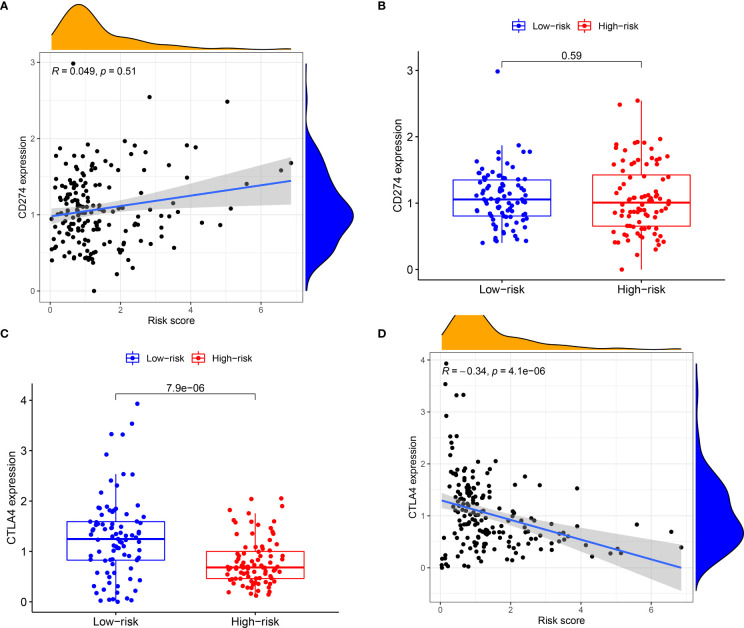
The relationship between IRGPRI score and the expression levels of PD-L1 or CTLA4. **(A)** Correlation analysis between IRGPRI and PD-L1. **(B)** PD-L1 in different IRGPRI subgroups. **(C)** CTLA4 expression in different IRGPRI subgroups. **(D)** Correlation analysis between IRGPRI and CTLA4 expression.

Subsequently, the association of IRGPRI score with CTLA4 expression and PD-L1 was explored. We found that the IRGPRI score was negatively correlated with CTLA4 (r = -0.34, *p* < 0.001), as shown in **Figures 5A**–**D**. Meanwhile, the association of IRGPRI score with marker genes of cell proliferation and migration was explored. We found that the IRGPRI score was positively correlated with PCNA (r = 0.25, p < 0.001), MKI67 (r = 0.38, p < 0.001) and MMP14 (r = 0.27, p < 0.001), as shown in **Figures S3A**–**F**.

### Immune cell infiltration and function in different IRGPRI subgroups

**Figure 6:**
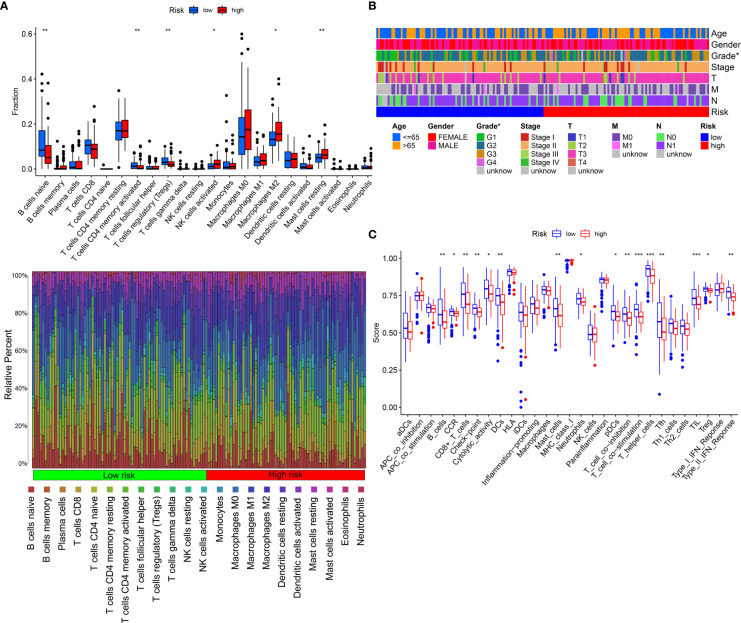
The landscape of the TME in PAAD and the characteristics of different IRGPRI subgroups. **(A)** The proportions of TME cells in different IRGPRI subgroups. **(B)** The IRGPRI grouping and proportions of TME cells for PAAD patients in the TCGA cohort. Age, Gender, Grade, Tumor stage, T, N, and survival M are shown as patient annotations. **(C)** The molecular and immune-related function of different IRGPRI subgroup. (*p < 0.05, **p< 0.01, ***p < 0.001).

To detect the constituents of immune cells in the IRGPRI subgroups, Wilcoxon test was performed to compare the distribution of immune cells in high- and low- IRGPRI subgroups. We found more abundant activated memory CD4+ T cells, B cell native and Tregs in the low IRGPRI subgroup, and more M2 macrophages, Mast cells resting and activated NK cells in the high IRGPRI subgroup (**Figure 6A**). **Figure 6B** displayed the features related to the immune landscape of different IRGPRI subgroups, including the clinicopathological features.

Then, we defined the molecular and immune function between different IRGPRI subgroups by certain gene signatures. There were more CD8+ T cells, checkpoints, T cell co-stimulation in the low IRGPRI subgroup (**Figure 6C**).

### Relationship between IRGPRI grouping and clinical and immune subtypes

**Figure 7:**
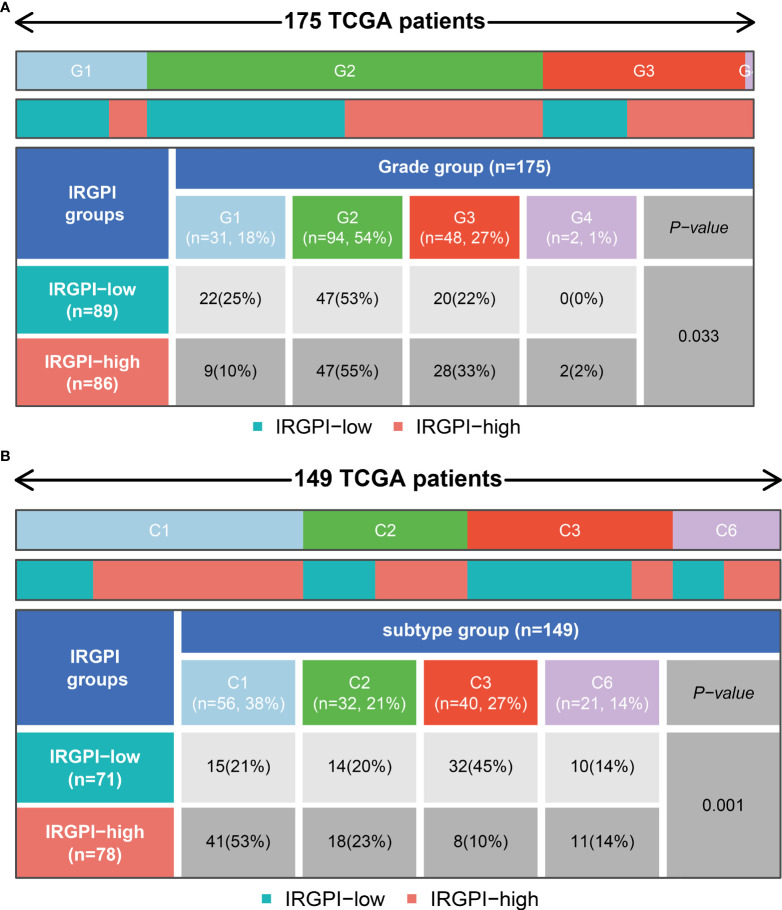
Relationship between IRGPRI grouping and clinical and immune subtypes.**(A)** Heat map and table showing the distribution of PAAD grade (G1, G2, G3 and G4) between the IRGPRI subgroups. **(B)** Heat map and table showing the distribution of PAAD immune subtypes (C1, C2, C3 and C6) between the IRGPRI subgroups.

We could find from **Figure 7A** and **Figure S4** that the proportion of the TNM stage was almost equally distributed between low- and high- IRGPRI groups, but there were more Grade 1 samples and fewer Grade 3/4 samples in the low IRGPRI group versus the high IRGPRI group (p = 0.033, chi-square test). In **Figure 7B**, more C1 immune subtypes were found in the high IRGPRI group and more C3 immune subtype were found in the low IRGPRI group (p = 0.001, chi-square test).

### Relationship between IRGPRI grouping and immunotherapy

**Figure 8:**
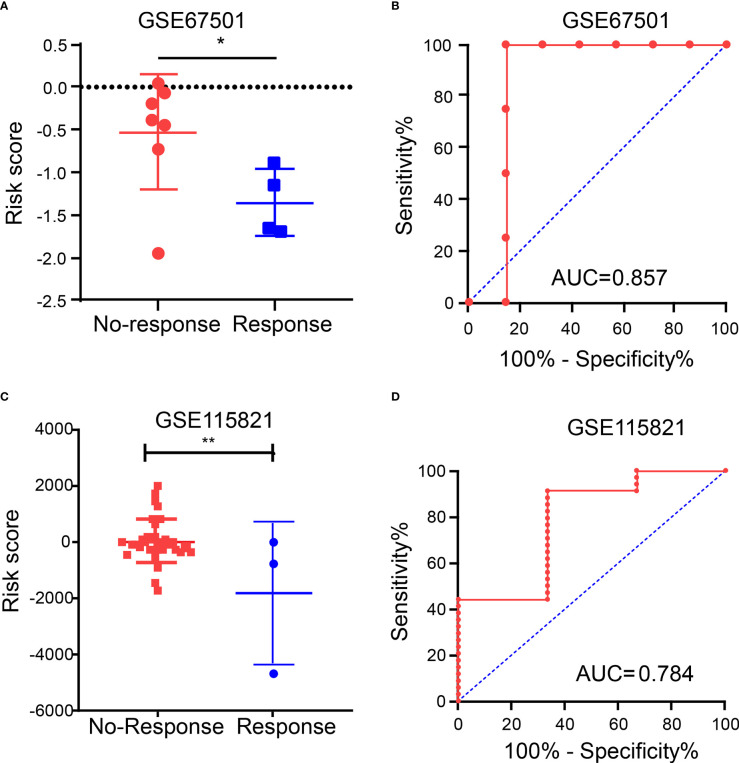
Immunotherapy efficacy in different IRGPRI subgroups. **(A)** The risk score in patients who did or did not respond to anti-PD-1 immunotherapy. **(B)** Diagnostic value of risk score by ROC curve in GSE67501. **(C)** The risk score in patients who did or did not respond to ICI therapy. **(D)** Diagnostic value of risk score by ROC curve in GSE115821. (*p < 0.05 and **p< 0.01).

Due to the lack of public data on PAAD immunotherapy, we can only select other tumor immunotherapy data to verify the predictive role of IRGPRI model. In order to further explore the predictive role of IRGPRI model in immunotherapy, we analyzed the expression data in samples from human renal cell carcinoma patients who did or did not respond to anti-PD-1 immunotherapy (GSE67501). The results showed that the risk score in patients who did not respond to anti-PD-1 immunotherapy (stable disease or progressive disease) was higher than it in patients who responded to anti-PD-1 immunotherapy (complete response or partial response) (**Figure 8A**). Moreover, we performed receiver operating characteristic (ROC) analysis to determine the diagnostic value of risk score in ICI therapy efficacy, and the area under the ROC curve is 0.857 (**Figure 8B**). Then we analyzed the expression data in metastatic melanoma samples from patients who did or did not respond to ICI therapy (GSE115821). The results also showed that the risk score in patients who did not respond to ICI therapy was higher than it in patients who responded to ICI therapy (**Figure 8C**). And the area under the ROC curve is 0.784 (**Figure 8D**). The above results suggest that IRGPRI may be a potential prediction model for predicting the efficacy of immunotherapy. The graphical abstract of our research is shown in the **Figure S5**.

## Discussion

The role of immune cells that constitute the TME in tumor progression has been recognized (20). Increasing evidence indicates that immune gene characteristics may be prognostic or predictive factors of PAAD (21, 22). Immunotherapy has been confirmed as an effective option for PAAD patients (23–25). Given that the immunosuppressive microenvironment of PAAD can affect the efficacy of immunotherapy (26–28), it is crucial to determine which patients will benefit most from these treatments. Although different prognostic markers for PAAD have been evaluated for multiple years, we still cannot find an effective biomarker to predict the prognostic outcomes of PAAD patients and the suitability for immunotherapy. This highlights the need to identify biomarkers for the PAAD prognosis and the efficacy of immunotherapy.

WGCNA is a virtual approach to assisting in identifying potential therapeutic targets or immune-related biomarkers. In this study, based on the PAAD immune gene dataset, WGCNA was used to determine 16 immune-related central genes that affected the OS of patients; and based on S100A16, CD40, VCAM1, TNFRSF4 and TRAF1 that were independent prognostic factors of OS, IRGPRI was constructed. IRGPRI has been proven to be an effective immune-related biomarker for the prognosis of PAAD. In TCGA and GEO arrays, the survival rate was lower in patients with high IRGPRI and higher in those with low IRGPRI.

IRGPRI is composed of five genomes: S100A16, CD40, VCAM1, TNFRSF4 and TRAF1. S100A16 has been shown to be associated with obese, type 2 diabetes mellitus and inflammation *via* calcium-dependent mechanism (29). Moreover, it has also been found that S100A16 is correlated with the occurrence and progression of many tumors (30–34). S100A16 enhances the progression and metastasis of PAAD *via* FGF19 mediated AKT and ERK1/2 pathway (30). The study of Gangping Tu, et al. showed that in comparison with the normal pancreas, S100A16 was highly expressed in tissues with PAAD, and the increase of its expression level may be correlated with an unfavorable prognosis of PAAD patients (35). CD40 is a cell surface member of the tumor necrosis factor (TNF) receptor superfamily. An active CD40 is closely related to the tumor immunity (36). VCAM1 expression is associated with the tumorigenesis and unfavorable prognosis of high-grade serous ovarian cancer (37). TNFRSF4 may be a promising immunotherapy target and prognostic biomarker for liver cancer (38). TRAF1 is important in the maintenance of immune function of CD8+T cells (39). In the computation formula of IRGPRI, the coefficient of S100A16, CD40 and VCAM1 is a positive number, while the coefficient of TNFRSF4 and TRAF1 is a negative number. Therefore, IRGPRI is negatively correlated with TNFRSF4 and TRAF1, while IRGPRI is positively correlated with S100A16, CD40 and VCAM1. In conclusion, IRGPRI is a biomarker that is associated with prognosis and tumor immunity.

We investigated gene mutations in different IRGPRI subgroups to further understand the immunological properties of IRGPRI subgroup. KRAS and TP53 mutations are more common in the high IRGPRI samples than those in the low IRGPRI samples. KRAS mutation is correlated with high circulating regulatory T cell levels, both of which indicate poorer prognosis in advanced PAAD patients (40). In addition, TP53 mutation is associated with more aggressive diseases and worse patient prognosis in various cancers (41, 42). KRAS, TP53, SMAD4 and CDKN2A are considered as the major drivers for the occurrence of PAAD. Among 71 patients who received adjuvant chemotherapy and radical surgery, those with less mutations in the four driver genes tended to obtain better outcomes (43). Therefore, as with our survival results, high IRGPRI group with high TP53 and KRAS mutations have a worse prognosis than low IRGPRI group with low TP53 and KRAS mutations.

Then, we will explore the correlation of IRGPRI with known predictive markers for immunotherapy, such as PD-L1 and CTLA4. In general, PD-L1+ and CTLA4+ tumors tend to respond better to immune checkpoint inhibitor therapy than negative tumors (44–46). Similar results were observed in PAAD, although IRGPRI scores were not strongly associated with PD-L1. However, we found a significant correlation between IRGPRI score and CTLA4, suggesting that CTLA4 may help explain why IRGPRI affected the prognosis of immunotherapy to a certain extent.

Understanding the TME may help find new methods for the treatment of PAAD, or modifying the TME may improve the effectiveness of immunotherapy. In the two IRGPRI subpopulations, there are differences in the constituent of certain immune cells and the activity of immune functions. CD8+ T cells, checkpoints, T cell co-stimulation are more active in the low IRGPRI group, while M2 macrophages are more common in the high IRGPRI group. Many studies have uncovered that intensive infiltration of T cells, especially cytotoxic CD8+ T cells, indicates a good prognosis (47–49). M2 macrophage is a major subtype of macrophages in most tumors, that promotes aggressive phenotype formation and tumor growth, and is associated with a poor prognosis of PAAD (50, 51). Meanwhile, there were more C1 immune subtypes in the high IRGPRI group and more C3 immune subtype in the low IRGPRI group. TCGA tumors can be clustered into six immune subtypes. C3 had the best prognosis, while C1 had less favorable outcomes (52). These conclusions also were supported by our study results. This implies that the high IRGPRI group has immunosuppressive characteristics, while the low IRGPRI group has better tumor immunity potential.

In order to further explore the predictive role of IRGPRI model in immunotherapy, we analyzed immunotherapy sequencing data. Since no sequencing cohort was found for the efficacy of PAAD immunotherapy, we analyzed renal cell carcinoma immunotherapy cohort (GSE67501) and metastatic melanoma immunotherapy cohort (GSE115821). The results showed that the risk score in patients who did not respond to ICI therapy was significantly higher than the risk score in patients who responded to ICI therapy. These results mean that IRGPRI may be a potential prediction model for predicting the efficacy of immunotherapy.

However, there are still some limitations in our study. First, a PAAD immunotherapy cohort is needed to verify the predictive role of IRGPRI in immunotherapy. Second, a prospective cohort study is needed to confirm the prognostic value of this model.

In conclusion, IRGPRI is an encouraging immune-related prognostic marker. IRGPRI may help identify molecular and immune features and predict the prognosis of PAAD patients. Additionally, IRGPRI may have predictive implication for immunotherapy, which should be further verified in further studies.

## Data availability statement

The datasets presented in this study can be found in online repositories. The names of the repository/repositories and accession number(s) can be found in the article/**supplementary material**.

## Author contributions

MS and YS made important contributions to the study conception and design. YS and RQ conducted data analysis and interpretation. All authors participated in the drafting and revision of the manuscript. All authors read, revised and approved this manuscript and agreed to be responsible for all aspects of the research to ensure the data accuracy and integrity of this work.

## Funding

This research was supported by Natural Science Research Plan of Huaian (No. HAB202022), National Natural Science Foundation of China (No. 81972719, 82003164), National science research in Universities of Jiangsu Province (No. 21KJA320008, 20KJB320032, 21KJB320021), Jiangsu Province Natural Science Foundation (No. BK20210910, BK20201012, BK20210913), Innovative and Entrepreneurial Doctor Project of Jiangsu Province (JSSCBS20211256), Key Research & Developement Plan of Xuzhou (No. KC18102), Science and Technology Project of Xuzhou (No. KC20126), Scientific Research Foundation of Xuzhou Medical University (No. D2019050).

## Conflict of interest

The authors declare that the research was conducted in the absence of any commercial or financial relationships that could be construed as a potential conflict of interest.

## Publisher’s note

All claims expressed in this article are solely those of the authors and do not necessarily represent those of their affiliated organizations, or those of the publisher, the editors and the reviewers. Any product that may be evaluated in this article, or claim that may be made by its manufacturer, is not guaranteed or endorsed by the publisher.

## Supplementary material

Supplementary Figure 1 Differentially expressed immune-related genes in PAAD. **(A)** Heatmap displaying all differentially expressed genes (DEGs) between PAAD samples (red) and para-cancer samples (blue) (p < 0.05, |log2FC| > 1). **(B)** Heatmap displaying immune-related DEGs between PAAD samples (red) and para-cancer samples (blue). **(C)** Gene Ontology (GO) enrichment analysis of the immune-related DEGs (p < 0.05). **(D)** Kyoto Encyclopedia of Genes and Genomes (KEGG) pathway analysis of the immune-related DEGs (p < 0.05).

Supplementary Figure 2 Determination of the soft-thresholding power in the WGCNA analysis. As seen from the graph, the optimal soft threshold for WGCNA was 6.

Supplementary Figure 3 The relationship between IRGPRI score and the expression levels of PCNA, MKI67 or MMP14. **(A)** PCNA in different IRGPRI subgroups. **(B)** Correlation analysis between IRGPRI and PCNA. **(C)** MKI67 expression in different IRGPRI subgroups. **(D)** Correlation analysis between IRGPRI and MKI67 expression. **(E)** MMP14 expression in different IRGPRI subgroups. **(F)** Correlation analysis between IRGPRI and MMP14 expression.

Supplementary Figure 4 Distribution of TNM stage subtypes in different IRGPRI subgroups. Heat map and table showing the distribution of PAAD stage (Stage 1, Stage 2, Stage 3 and Stage 4) between the IRGPRI subgroups.

Supplementary Figure 5 Graphical Abstract.
