# Supplementary figures and images for: An immune-related gene prognostic risk index for pancreatic adenocarcinoma
Source: Front Immunol. 2022 Jul 26;13:945878. doi: 10.3389/fimmu.2022.945878 (PMC9360334; doi:10.3389/fimmu.2022.945878)

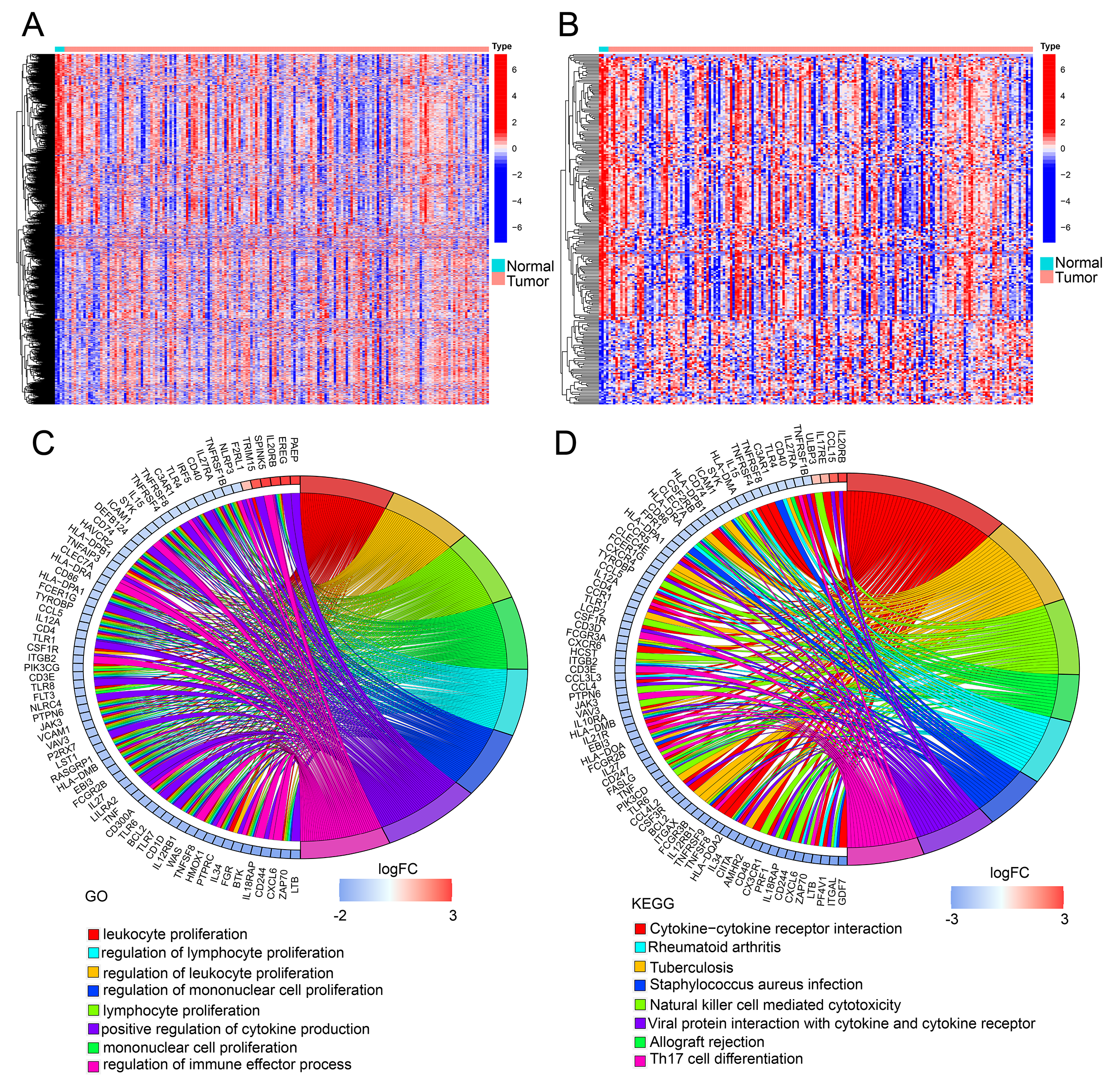

Supplement: Supplementary Figure 1 — Differentially expressed immune-related genes in PAAD. (A) Heatmap displaying all differentially expressed genes (DEGs) between PAAD samples (red) and para-cancer samples (blue) (p < 0.05, |log2FC| > 1). (B) Heatmap displaying immune-related DEGs between PAAD samples (red) and para-cancer samples (blue). (C) Gene Ontology (GO) enrichment analysis of the immune-related DEGs (p < 0.05). (D) Kyoto Encyclopedia of Genes and Genomes (KEGG) pathway analysis of the immune-related DEGs (p < 0.05). [file Image_1.tif]

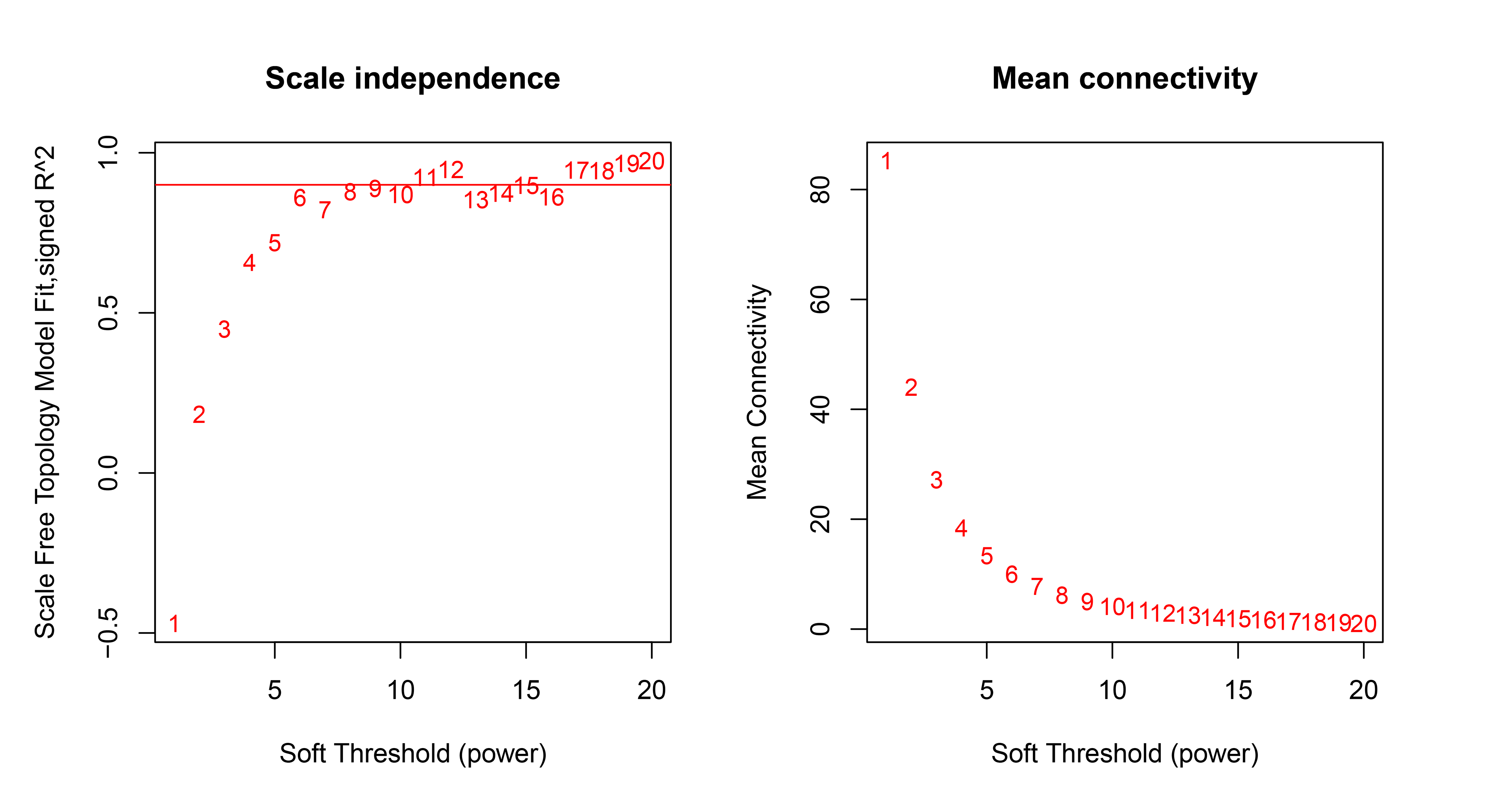

Supplement: Supplementary Figure 2 — Determination of the soft-thresholding power in the WGCNA analysis. As seen from the graph, the optimal soft threshold for WGCNA was 6. [file Image_2.tif]

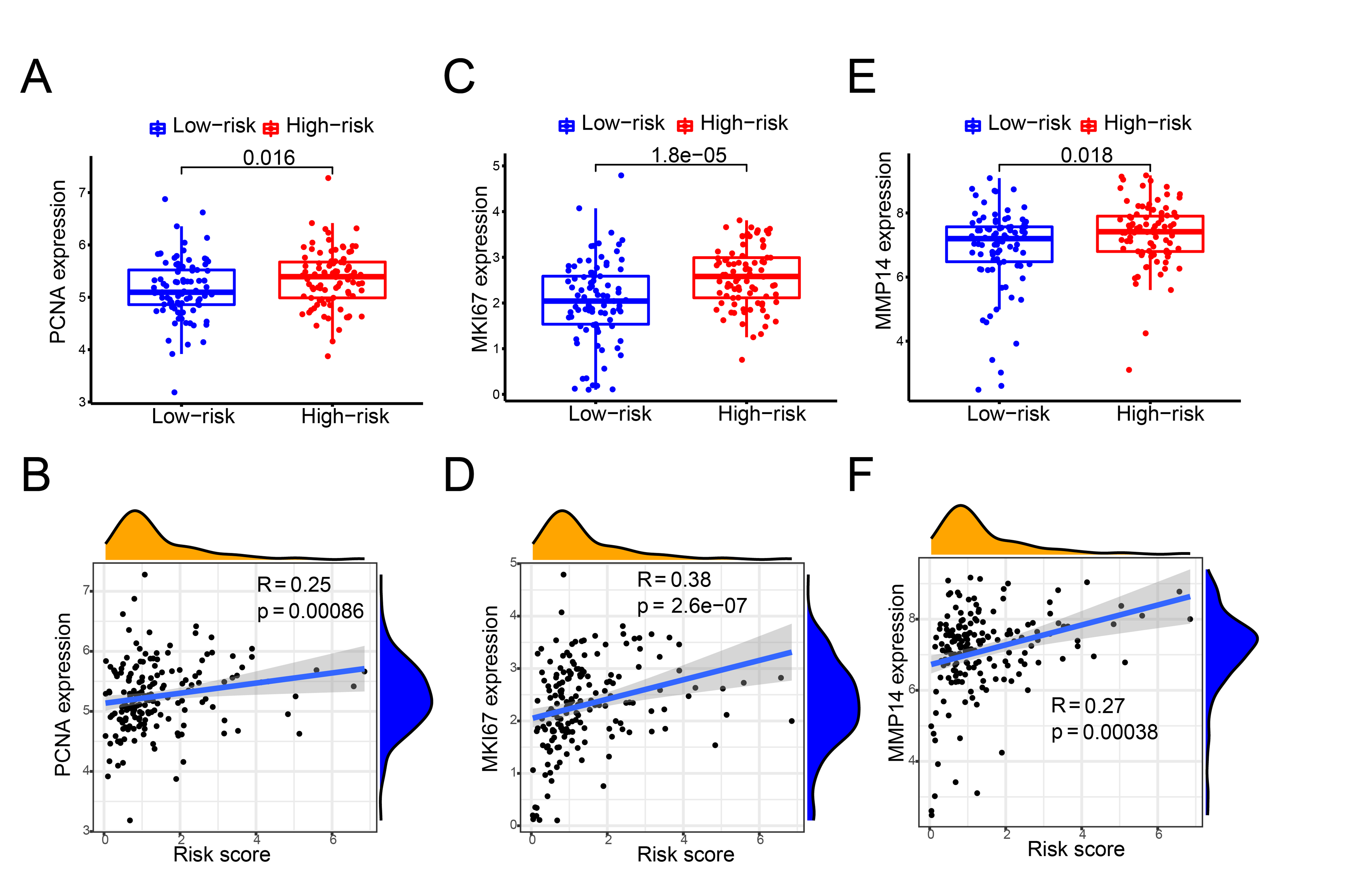

Supplement: Supplementary Figure 3 — The relationship between IRGPRI score and the expression levels of PCNA, MKI67 or MMP14. (A) PCNA in different IRGPRI subgroups. (B) Correlation analysis between IRGPRI and PCNA. (C) MKI67 expression in different IRGPRI subgroups. (D) Correlation analysis between IRGPRI and MKI67 expression. (E) MMP14 expression in different IRGPRI subgroups. (F) Correlation analysis between IRGPRI and MMP14 expression. [file Image_3.tif]

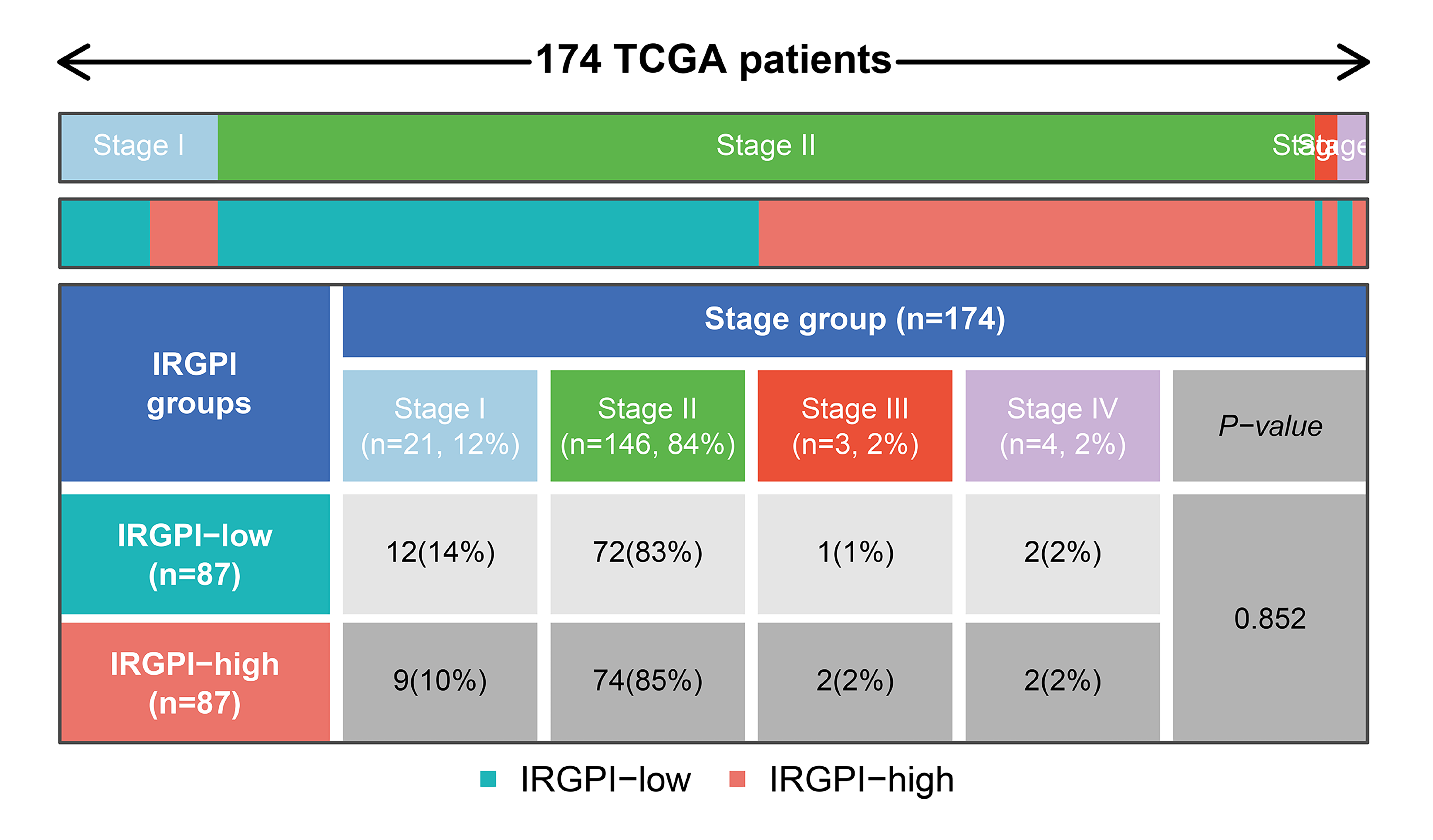

Supplement: Supplementary Figure 4 — Distribution of TNM stage subtypes in different IRGPRI subgroups. Heat map and table showing the distribution of PAAD stage (Stage 1, Stage 2, Stage 3 and Stage 4) between the IRGPRI subgroups. [file Image_4.tif]

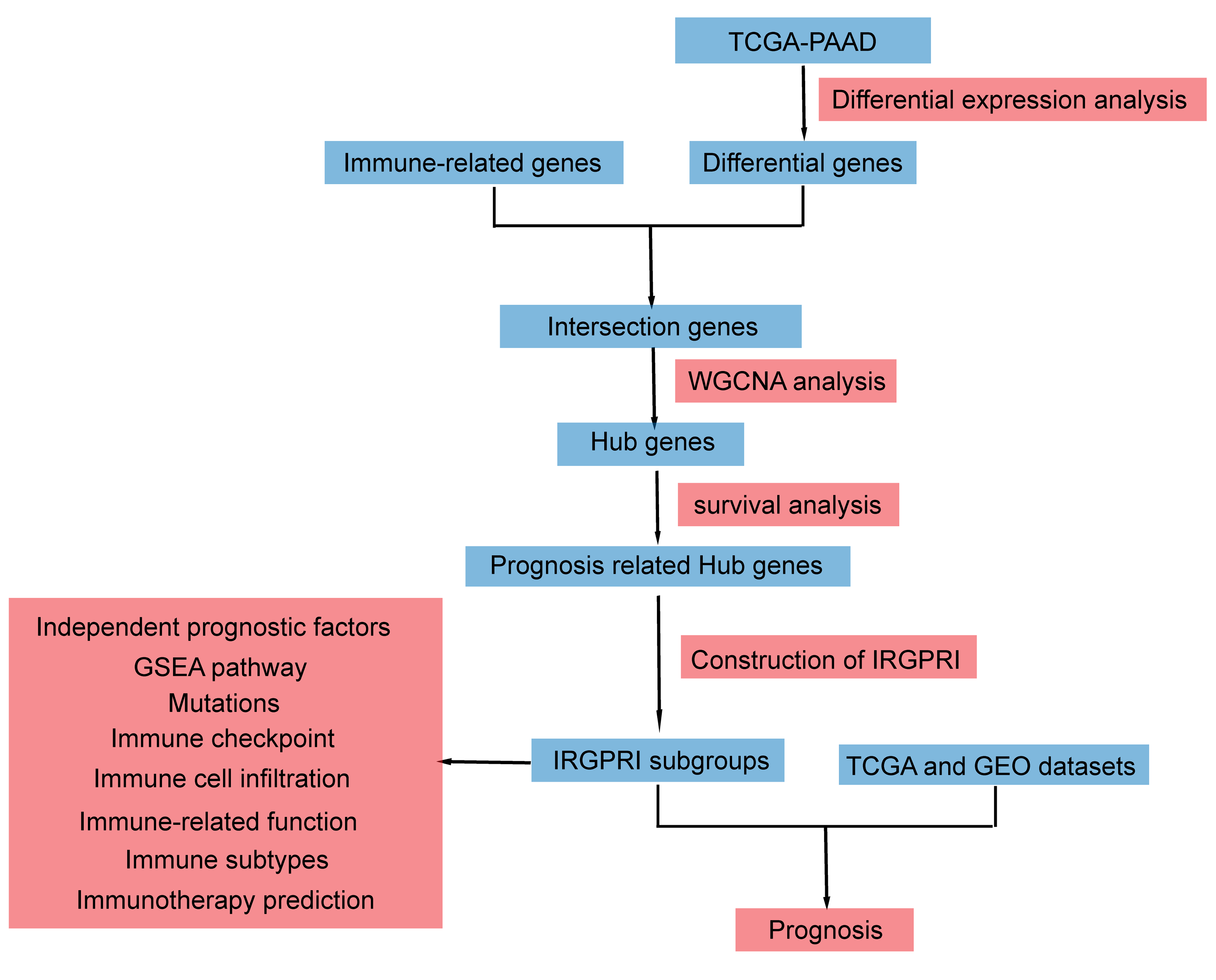

Supplement: Supplementary Figure 5 — Graphical Abstract. [file Image_5.tif]
