# Supplementary material for: An immune-related gene prognostic risk index for pancreatic adenocarcinoma
Source: Front Immunol. 2022 Jul 26;13:945878. doi: 10.3389/fimmu.2022.945878 (PMC9360334; doi:10.3389/fimmu.2022.945878)
Supplement: Supplementary file 7 [file Table_2.docx]

| **Table S2.** The coefficient of S100A16, CD40, VCAM1, TNFRSF4 and TRAF1 genes | | |
| --- | --- | --- |
| Id | Coefficient |  |
| S100A16 | 0.386448799 |  |
| CD40 | 0.529324919 |  |
| VCAM1 | 0.265544092 |  |
| TNFRSF4 | -0.528702301 |  |
| TRAF1 | -1.015782955 |  |
