# Supplementary material for: An immune-related gene prognostic risk index for pancreatic adenocarcinoma
Source: Front Immunol. 2022 Jul 26;13:945878. doi: 10.3389/fimmu.2022.945878 (PMC9360334; doi:10.3389/fimmu.2022.945878)
Supplement: Supplementary file 8 [file Table_3.docx]

| **Table S3.** The risk characteristics adjusted by all factors | | | | |
| --- | --- | --- | --- | --- |
| Id | HR | HR.95L | HR.95H | *p*-value |
| Age | 1.029 | 1.007 | 1.051 | 0.010 |
| Gender | 0.771 | 0.506 | 1.176 | 0.227 |
| Grade | 1.146 | 0.840 | 1.563 | 0.388 |
| Stage | 1.196 | 0.780 | 1.833 | 0.412 |
| riskScore | 1.536 | 1.337 | 1.764 | <0.001 |
